# Supplementary material for: Backbone amides are determinants of Cl− selectivity in CLC ion channels
Source: Nat Commun. 2022 Dec 6;13:7508. doi: 10.1038/s41467-022-35279-1 (PMC9726985; doi:10.1038/s41467-022-35279-1)
Supplement: Supplementary file 1 — Supplementary Information [file 41467_2022_35279_MOESM1_ESM.pdf]

Supplementary Information

Backbone amides are determinants of Cl^{-} selectivity in CLC ion channels

Lilia Leisle[1], Kin Lam[2], Sepehr Dehghani-Ghahnaviyeh[2], Eva Fortea[1,3], Jason D. Galpin[4], Christopher A. Ahern[4], Emad Tajkhorshid[2] and Alessio Accardi[1,3,5]

[1] Department of Anesthesiology, Weill Cornell Medical College, New York, NY, USA,

[2] Theoretical and Computational Biophysics Group, NIH Center for Macromolecular Modeling and Bioinformatics, Beckman Institute for Advanced Science and Technology, Department of Biochemistry, and Center for Biophysics and Quantitative Biology, University of Illinois at Urbana-Champaign, Urbana, Illinois 61801, USA

[3] Department of Physiology and Biophysics, Weill Cornell Medical College, New York, NY, USA;

[4] Department of Molecular Physiology and Biophysics, University of Iowa, Iowa City, IA, USA

[5] Department of Biochemistry, Weill Cornell Medical College, New York, NY, USA.

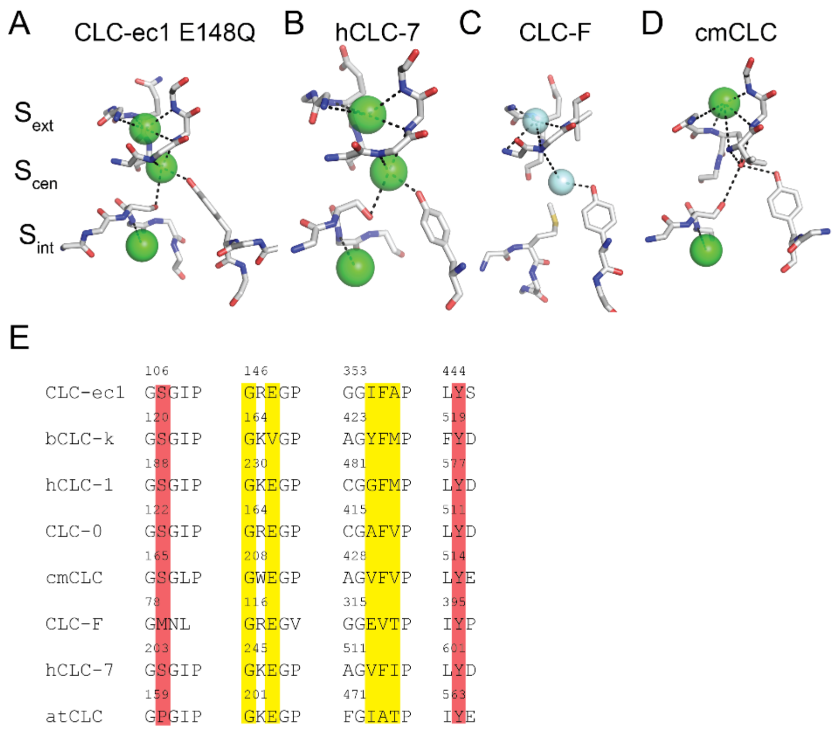

Supplementary Figure 1. Structural and sequence conservation of the CLC ion pathway. (A-D) Close up view of the Cl^- permeation pathway in E148Q CLC-ec1(PDB: 1OTU, A), hCLC-7 (PDB: 7JM7, B), CLC-F (PDB: 6D0J, C) and cmCLC (PDB: 3ORG, D). Bound Cl^- (F^-) ions are shown as green (cyan) spheres. Dashed black lines indicate hydrogen bonds between the anions and the protein. (E) Sequence alignment of the ion coordination motifs in CLC channels (bCLC-k, hCLC-1 and CLC-0) and transporters (CLC-ec1, hCLC-7, cmCLC, CLC-F, and atCLC-a). Yellow and red shading respectively indicate residues mediating anion coordination via backbone amides or side chains.

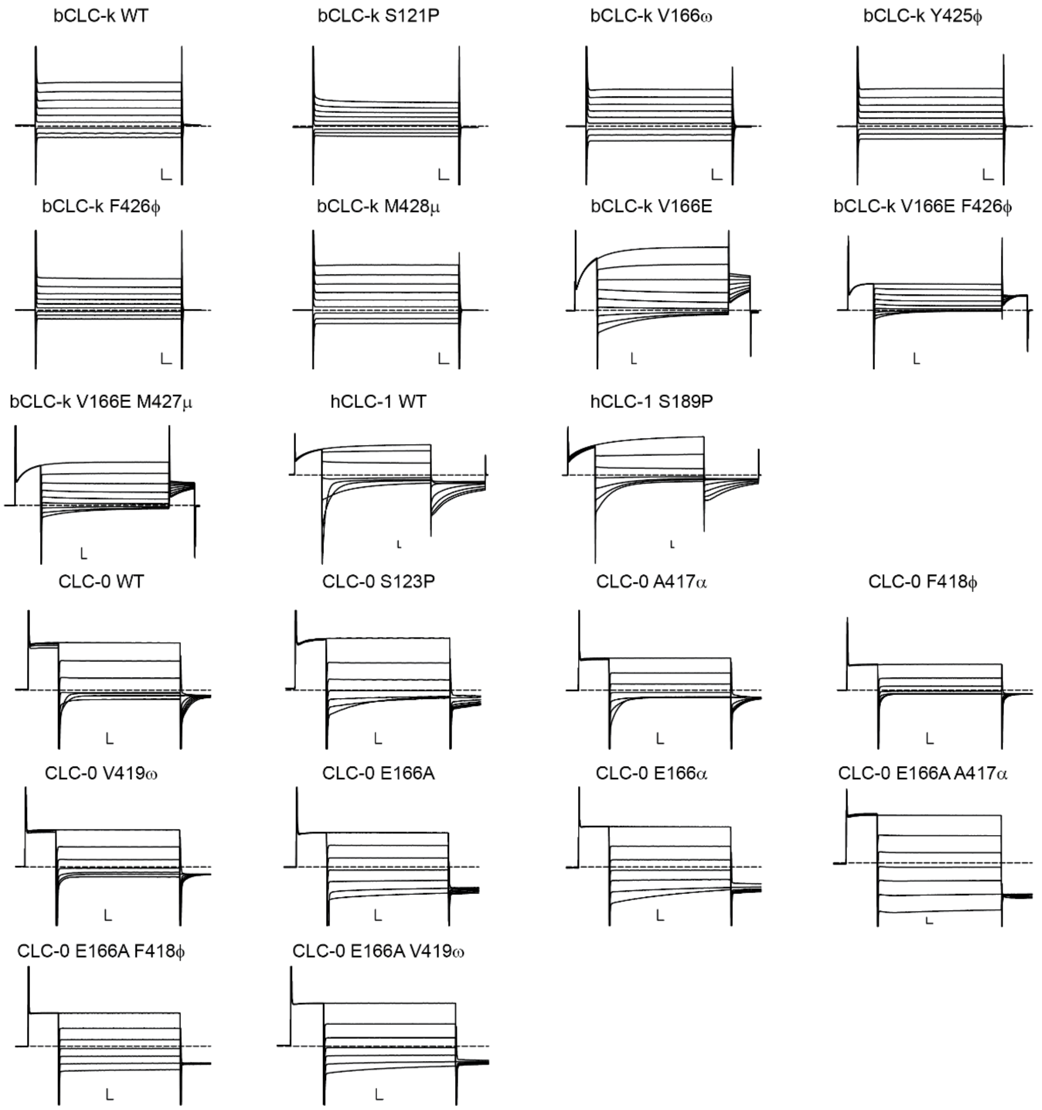

Supplementary Figure 2. Representative currents of all WT and mutant constructs of bCLC-k, CLC-1 and CLC-0 channels. Stimulation protocols are described in the methods section. Dashed lines indicate the 0 current level. In all cases scale bars indicate 2 μA and 10 ms. Traces for CLC-0 (WT and S123P), bCLC-k (WT and S121P) and hCLC-1 (WT and S189P) are the same as shown in Fig. 1. Raw data for Supp. Fig. 2 is included in the Source Data Files.

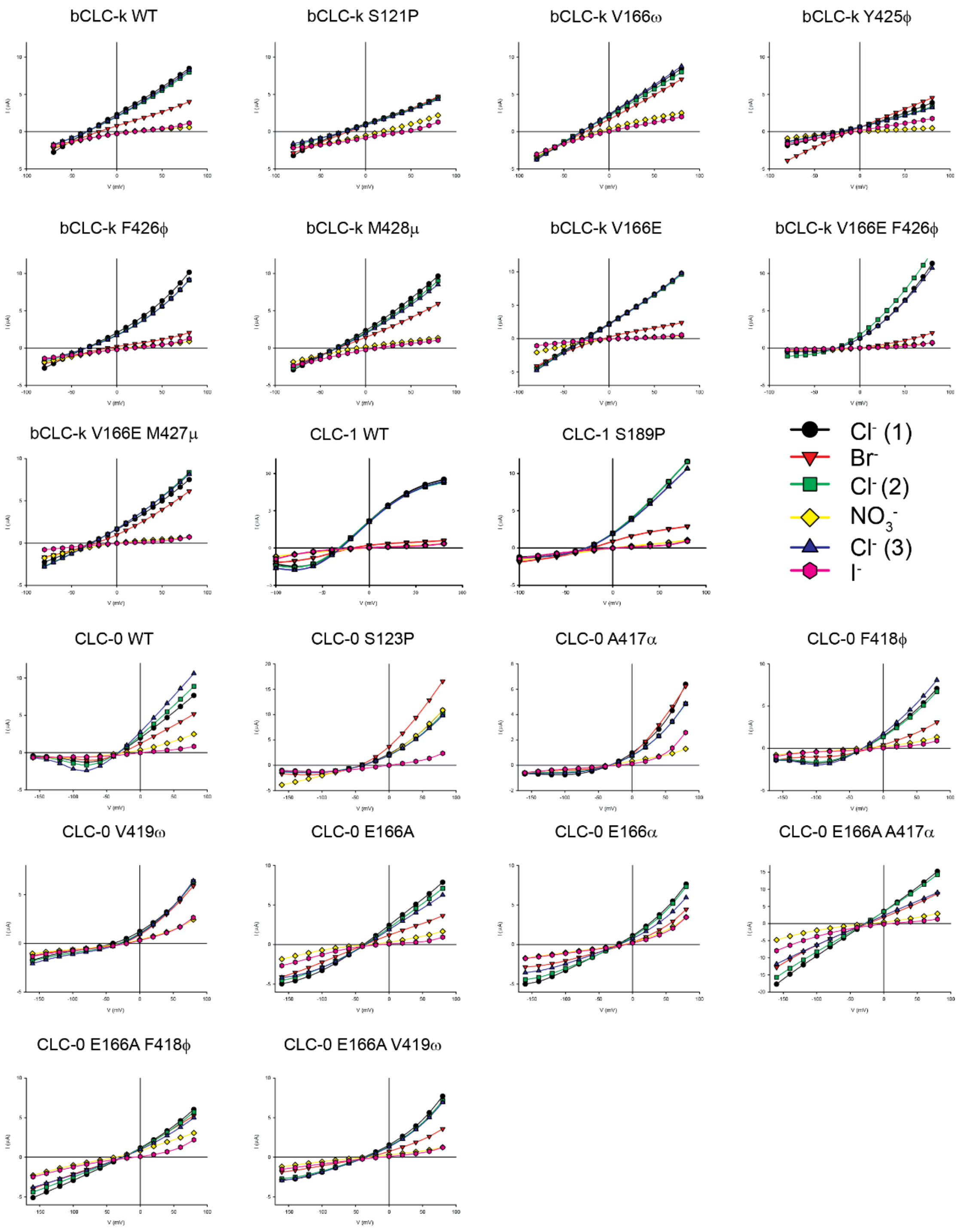

Supplementary Figure 3. I-V relationships for all WT and mutant constructs of bCLC-k, CLC-1 and CLC-0 channels. Representative steady state I-V relationships measured during ion substitution experiments Cl^-(1) (black circles), Br^- (red triangles), Cl^-(2) (green squares), NO_3^- (yellow diamond), Cl^-(3) (blue inverted triangle), I^- (pink hexagon). Experiments shown are for a single cell and un-normalized.

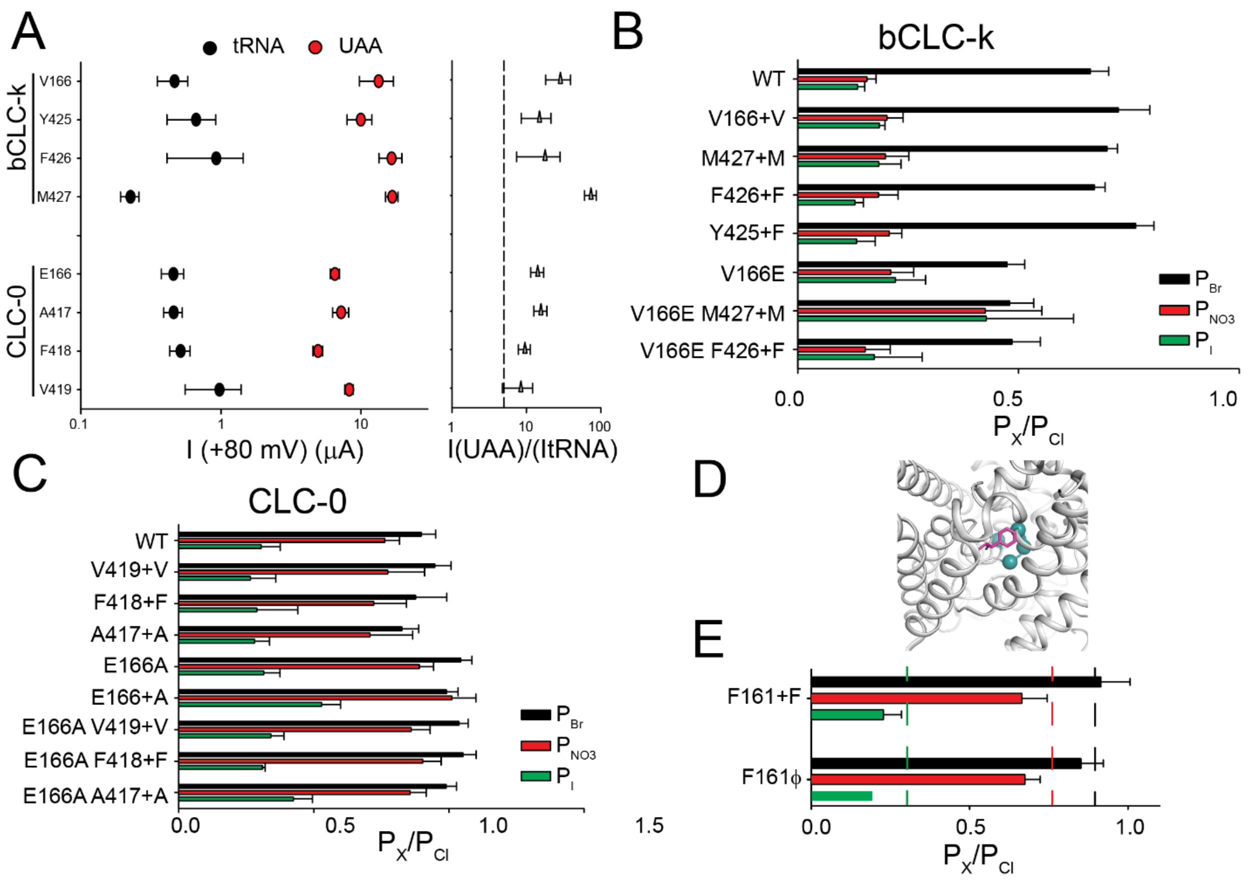

Supplementary Figure 4. Application of the nonsense suppression method to CLC Cl^{-} channels. (A) (right panel) Site-specific incorporation of non-canonical amino acids into the bCLC-k and CLC-0 channels is efficient and yields robust currents. Mean current I at +80 mV in *Xenopus laevis* oocytes for bCLC-k V166X, Y425X, F426X and M427X and CLC-0 E166X, A417X, F418X, V419X co-injected with empty tRNA (black) or misacylated tRNA loaded with the corresponding α-hydroxy acid (red). (left panel) The ratio I(UAA)/I(tRNA) was calculated from the mean values of I(UAA) and I(tRNA) at +80 mV. Errors were propagated. A threshold of I(UAA)/I(tRNA) >5 is imposed for specific incorporation efficiency (dashed line). (B-C) Incorporation of conventional aminoacids in bCLC-k (B) and CLC-0 (C) channels using the nonsense suppression method results in WT-like ion selectivity profiles. (D) Close up view of the outer vestibule of the bCLC-k pore highlighting the position of F161 (pink stick), and the Cα atoms of V166, Y425, F426 and M427 (cyan spheres). (E) Incorporation of Phe or φ at position F161 in CLC-0 results in WT-like ion selectivity. All values are reported as mean ± St.Dev of N>7 repeats from at least 3 independent oocyte batches. Individual data points for experiments in panels B-C-E are reported in Supp. Fig. 5. Raw data for Supp. Fig. 4B-C is included in the Source Data Files.

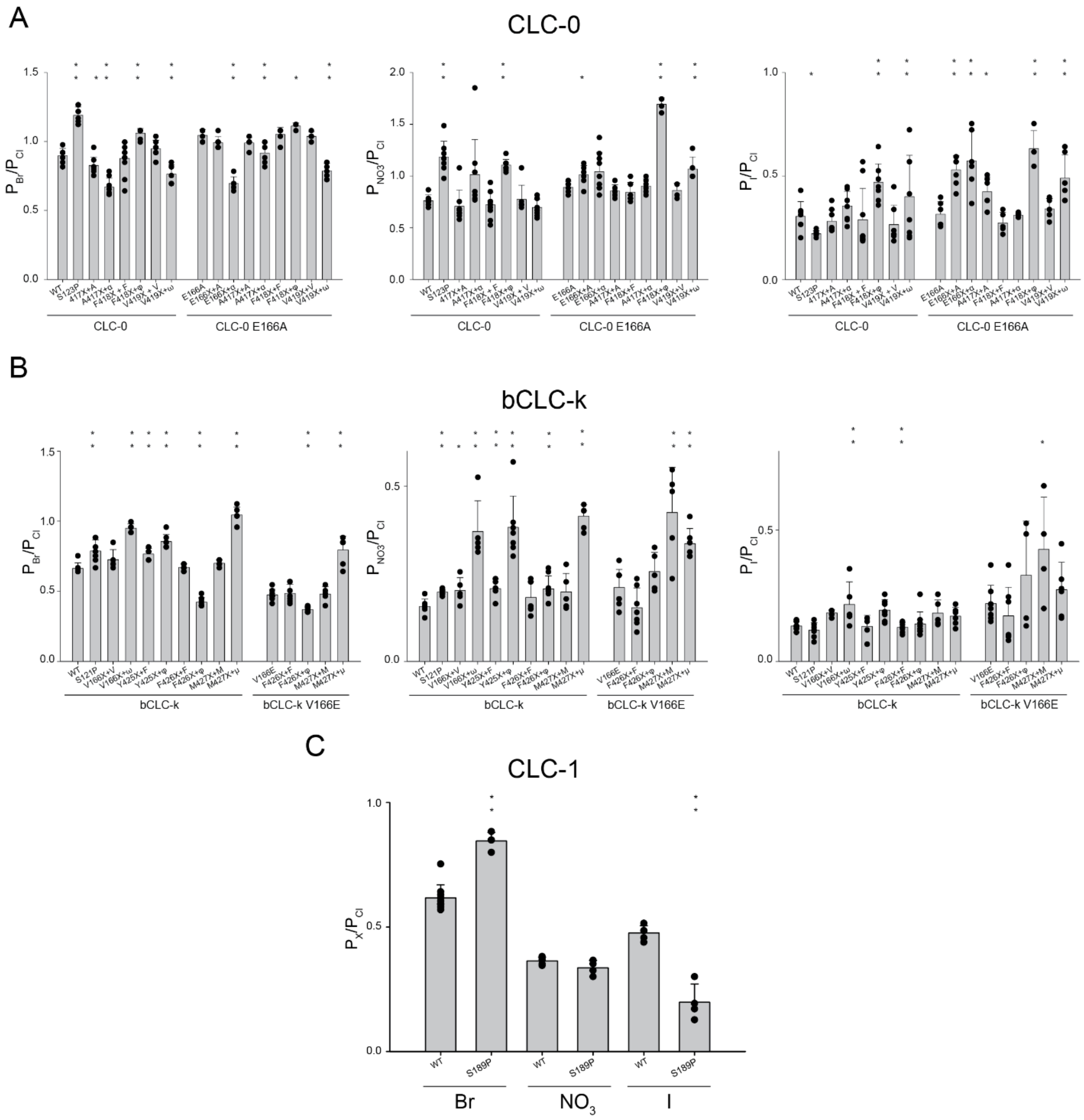

Supplementary Figure 5. Statistical evaluation of effects of main and side chain mutation on the anion selectivity of CLC-0, bCLC-k and CLC-1. (A-C) Permeability ratios P_{Br}/P_{Cl} (left panels), P_{NO3}/P_{Cl} (central panels) and P_{I}/P_{Cl} (right panels) of WT and mutant CLC-0 (A) and bCLC-k (B) and CLC-1 (C). Nomenclature of α-hydroxy acid substitutions is explained in Methods. Bars represent the Mean ± St.Dev. of n>4 repeats from N≥3 independent oocyte batches (data is the same as shown in Fig. 1, 2, 4 and in Supplementary Fig. 4) and circles indicate values from individual experiments. We compared the effects of each α-hydroxy substitution to its parent construct. Thus, each single mutant was compared to the WT channel constructs whereas each double mutant was compared to the corresponding E166A or V166E mutants for CLC-0 and bCLC-k, respectively. The threshold for significance of the one-sided Student's t-test was assigned using a threshold of $p=0.05$ and a Bonferroni correction. Thus, the significance thresholds are: CLC-0 WT $p<0.007$, CLC-0 E166A $p<0.006$; bCLC-k WT $p<0.0055$; bCLC-k V166E $p< 0.01$ and CLC-1 $p<0.05$. Values and p-values are reported in Supplementary Table 1. Raw data for Supp. Fig 5 is included in the Source Data Files.

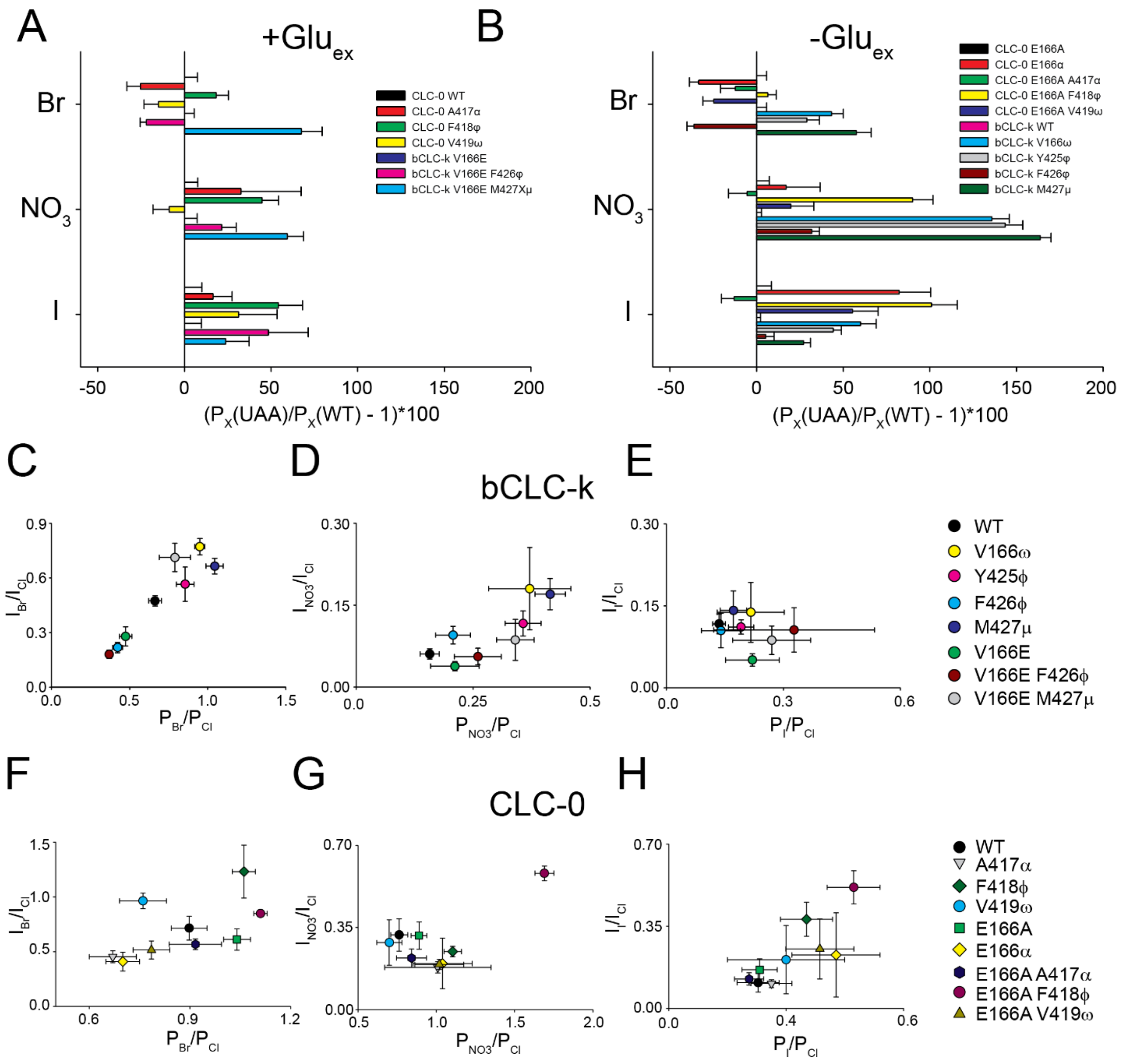

Supplementary Figure 6. Effects of backbone amide substitutions on ion permeability and ion conductivity. A-B) Normalized effects on P_{Br}, P_{NO3} and P_I elicited by backbone mutations in CLC channel constructs with (A) and without (B) a negatively charged side chain at Glu_{ex}. To compare effects due to backbone substitutions, values of single mutants were normalized to those of the WT parent channel, and of double mutants were normalized to those of the parent single Glu_{ex} mutant (V166E for bCLC-k and E166A for CLC-0). Error bars represent the propagation of the errors of the means. C-H) Relationship between the effects of backbone mutations in bCLC-k (C-E) and CLC-0 (F-H) on permeability and conduction of Br^- (C, F), NO_3^- (D, G) and I^- (E, H). Raw data for Supp. Fig. 7 is included in the Source Data Files.

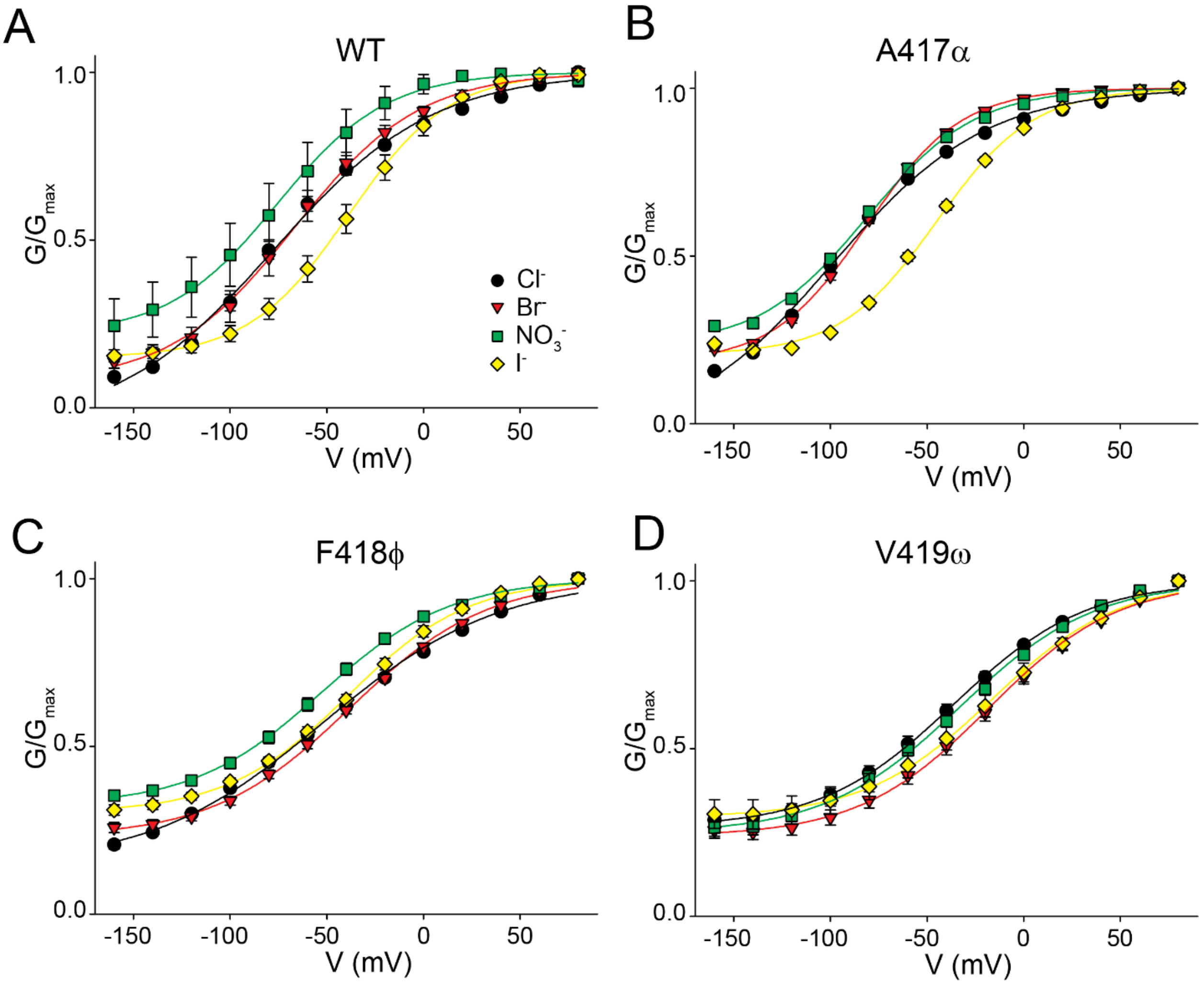

Supplementary Figure 7. Backbone mutations affect the anion-dependent modulation of CLC-0 fast gating. A-D) Average fast gate G-V relationships for WT (A), A417α (B), F418φ (C) and V419ω (D) measured in Cl^- (black circles), Br^- (red triangles), NO_3^- (green squares) and I^- (yellow diamond). Data are Mean ± St.Dev. of n>5 repeats from N≥3 independent oocyte batches, individual data points are shown as cyan circles.

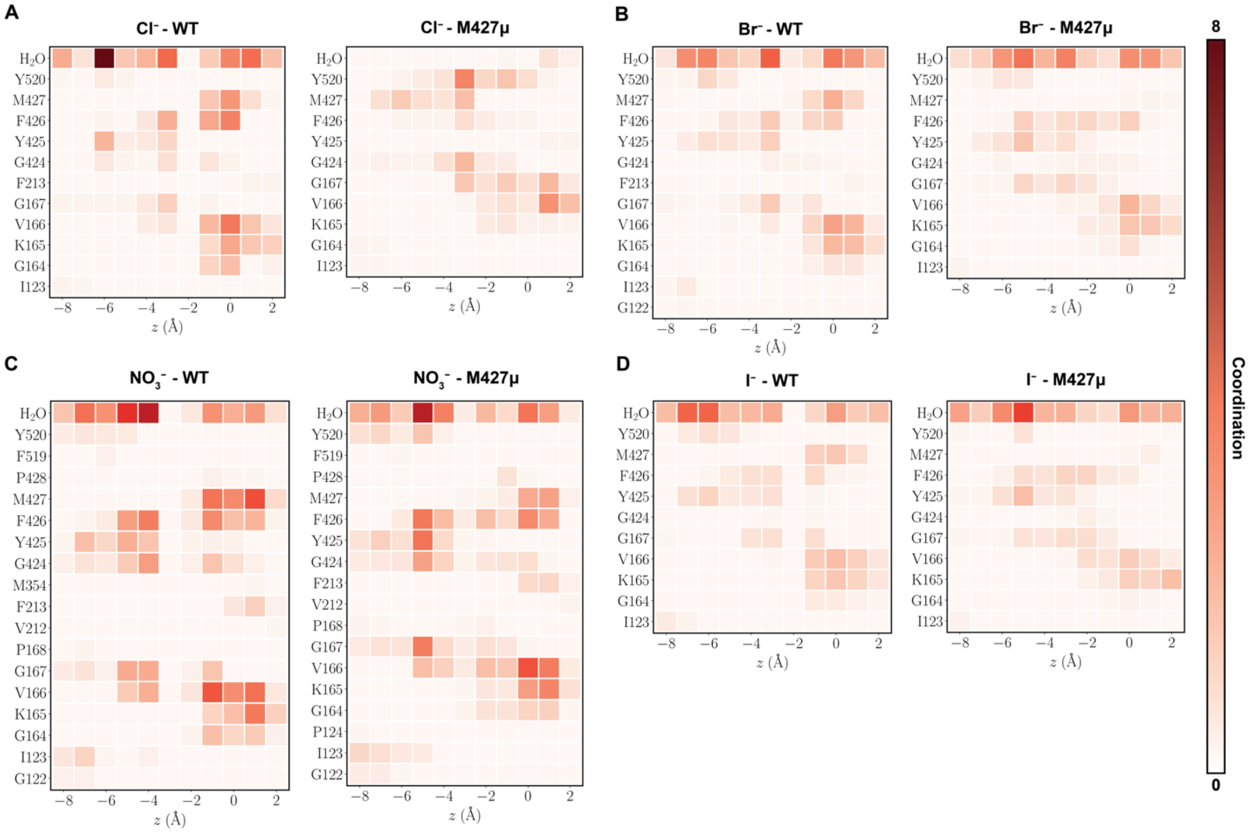

Supplementary Figure 8. Coordination of anions along the permeation pathway in WT and M427μ channels. A-D) show the coordination number of Cl^- (A), Br^-(B), NO_3^-(C), and I^- (D) by protein residues and water, respectively. The coordination numbers, averaged over the trajectories for each ion, are calculated for ions located in the range of $-8 \leq z \leq 2$ Å (with S_{ext} at z = 0), using a colormap shown in different shades of red. Different patterns of coordination contribute to different PMF profiles calculated for each ion.

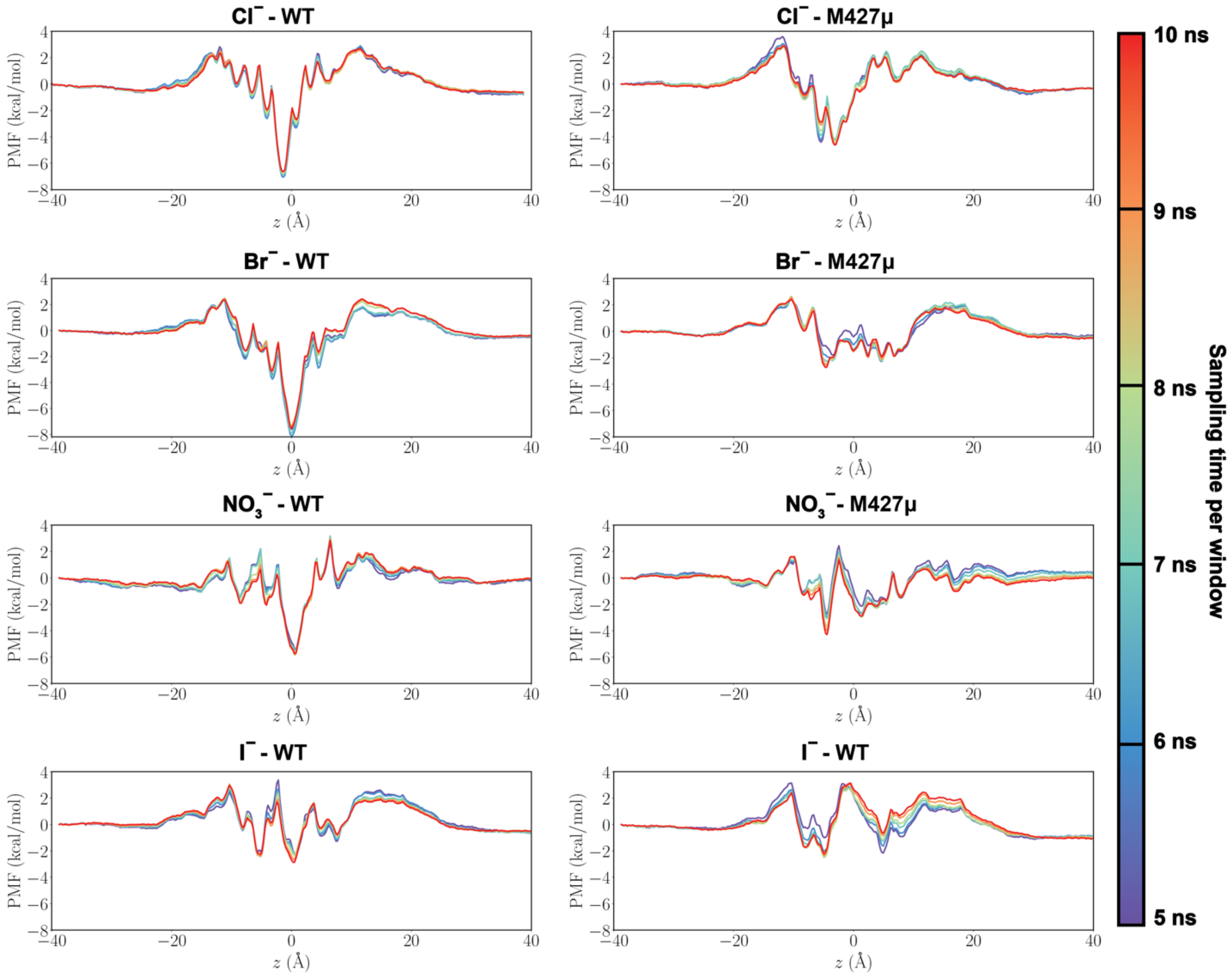

Supplementary Figure 9. Convergence of calculated PMF profiles. To examine the convergence of the PMFs, each curve is constructed for varying sampling times ranging between 5 ns (blue) and 10 ns (red) with 1 ns intervals, for both WT and M427μ systems. As shown, the PMFs remain unchanged after 9 ns of sampling.

Channel	Construct	P_{Br}/P_{Cl}	P value		n	P_{NO3}/P_{Cl}	P value	n		P_{I}/P_{Cl}	P value		n
**CLC-0**	WT	0.90±0.05			7	0.76±0.05		7		0.30±0.07			7
	S123P	1.19±0.06	$1.7*10^{-7}$	*	8	1.19±0.15	$8.8*10^{-6}$	8	*	0.22±0.02	0.01		7
	A417X+A	0.83±0.06	0.03		8	0.71±0.16	0.39	8		0.28±0.06	0.48		8
	A417X+α	0.67±0.07	$5.8*10^{-6}$	*	8	1.01±0.34	0.08	9		0.35±0.07	0.19		8
	F418X+F	0.88±0.11	0.67		10	0.72±0.12	0.42	10		0.29±0.15	0.80		9
	F418X+φ	1.06±0.03	$7.2*10^{-6}$	*	8	1.10±0.06	$2.0*10^{-8}$	8	*	0.47±0.09	$1.8*10^{-3}$	*	8
	V419X+V	0.95±0.06	0.12		7	0.77±0.13	0.84	7		0.26±0.09	0.39		7
	V419X+ω	0.76±0.07	$8.1*10^{-4}$	*	9	0.70±0.08	0.08	9		0.40±0.20	0.25		8
**CLC-0 E166A**	E166A	1.04±0.04			6	0.89±0.05		6		0.32±0.06			6
	E166X+A	0.99±0.04	0.05		7	1.01±0.09	0.01	7		0.53±0.07	$1.1*10^{-4}$	*	7
	E166X+α	0.69±0.05	$9.2*10^{-8}$	*	6	1.04±0.19	0.08	9		0.57±0.14	$2.4*10^{-3}$	*	6
	A417X+A	0.99±0.04	0.04		6	0.86±0.06	0.31	6		0.42±0.07	0.02		6
	A417X+α	0.92±0.08	$5.0*10^{-3}$	*	6	0.84±0.10	0.30	6		0.27±0.05	0.26		5
	F418X+F	1.05±0.05	0.78		6	0.90±0.07	0.72	6		0.31±0.01	0.86		6
	F418X+φ	1.11±0.02	0.02		4	1.69±0.06	$2.0*10^{-8}$	4	*	0.63±0.09	$1.2*10^{-4}$	*	4
	V419X+V	1.04±0.03	0.78		4	0.86±0.07	0.44	4		0.34±0.05	0.46		5
	V419X+ω	0.79±0.05	$1.1*10^{-6}$	*	7	1.06±0.12	$8.7*10^{-3}$	5	*	0.49±0.11	$4.4*10^{-3}$	*	9
**bCLC-k**	WT	0.66±0.04			7	0.16±0.02		7		0.14±0.02			7
	S121P	0.79±0.08	$3.4*10^{-3}$	*	7	0.20±0.01	$3.5*10^{-4}$	7	*	0.12±0.03	0.20		7
	V166X+V	0.73±0.07	0.08		5	0.20±0.04	0.02	5		0.18±0.01	$2.1*10^{-4}$	*	5
	V166X+ω	0.95±0.03	$1.3*10^{-7}$	*	5	0.37±0.09	$8.7*10^{-5}$	5	*	0.22±0.09	0.03		5
	Y425X+F	0.77±0.04	$1.6*10^{-3}$	*	5	0.21±0.03	$4.5*10^{-3}$	5	*	0.13±0.04	0.91		5
	Y425X+φ	0.86±0.05	$5.4*10^{-6}$	*	7	0.38±0.09	$2.8*10^{-}5$	7	*	0.19±0.04	$3.3*10^{-3}$	*	7
	F426X+F	0.67±0.02	0.63		7	0.18±0.04	0.20	7		0.13±0.02	0.56		7
	F426X+φ	0.42±0.03	$2.5*10^{-9}$	*	9	0.21±0.04	$7.80*10^{-3}$	8	*	0.14±0.05	0.71		9
	M427X+M	0.70±0.02	0.09		5	0.20±0.05	0.09	5		0.18±0.05	0.04		5
	M427X+μ	1.05±0.06	$1.8*10^{-8}$	*	6	0.41±0.03	$2.3*10^{-9}$	6	*	0.17±0.03	0.03		6
**bCLC-k V166E**	V166E	0.47±0.04			8	0.21±0.05		8		0.22±0.07			8
	F426X+F	0.49±0.06	0.68		7	0.15±0.06	0.06	7		0.17±0.11	0.33		7
	F426X+φ	0.37±0.02	$2.0*10^{-4}$	*	5	0.26±0.05	0.16	5		0.33±0.21	0.20		4
	M427X+M	0.48±0.06	0.84		5	0.42±0.13	$1.3*10^{-3}$	5	*	0.43±0.20	0.02		4
	M427X+μ	0.79±0.10	$2.5*10^{-6}$	*	6	0.34±0.04	$4.5*10^{-4}$	6	*	0.27±0.10	0.28		6
**hCLC-1**	WT	0.62±0.05			10	0.36±0.02		5		0.48±0.03			5
	S189P	0.85±0.03	$4.2*10^{-6}$	*	4	0.34±0.03	0.11	4		0.20±0.07	$1.0*10^{-4}$	*	4

Supplementary Table 1. Summary of effects of pore mutants on the ion selectivity of CLC-0, bCLC-k and hCLC-1. Data is reported as Mean ± St.Dev. of n repeats from N≥3 independent oocyte batches. Statistical significance was evaluated with one-sided Student's t-test with a Bonferroni correction. The significance thresholds are: CLC-0 WT $p<0.007$, CLC-0 E166A $p<0.006$; bCLC-k WT $p<0.0055$; bCLC-k V166E $p< 0.01$ and CLC-1 $p<0.05$. Each mutant construct was compared to its parent construct, thus single mutants were compared to WT channels and double mutants were compared to E166A and V166E for CLC-0 and bCLC-k, respectively.
