# Supplementary material for: Backbone amides are determinants of Cl− selectivity in CLC ion channels
Source: Nat Commun. 2022 Dec 6;13:7508. doi: 10.1038/s41467-022-35279-1 (PMC9726985; doi:10.1038/s41467-022-35279-1)
Supplement: Supplementary file 3 — Reporting Summary [file 41467_2022_35279_MOESM3_ESM.pdf]

nature portfolio

Corresponding author(s): Alessio Accardi

Last updated by author(s): Nov 13, 2022

Reporting Summary

Nature Portfolio wishes to improve the reproducibility of the work that we publish. This form provides structure for consistency and transparency in reporting. For further information on Nature Portfolio policies, see our Editorial Policies and the Editorial Policy Checklist.

Statistics

For all statistical analyses, confirm that the following items are present in the figure legend, table legend, main text, or Methods section.

n/a	Confirmed	
☐	☒	The exact sample size (*n*) for each experimental group/condition, given as a discrete number and unit of measurement
☐	☒	A statement on whether measurements were taken from distinct samples or whether the same sample was measured repeatedly
☐	☒	The statistical test(s) used AND whether they are one- or two-sided<br>*Only common tests should be described solely by name; describe more complex techniques in the Methods section.*
☒	☐	A description of all covariates tested
☒	☐	A description of any assumptions or corrections, such as tests of normality and adjustment for multiple comparisons
☐	☒	A full description of the statistical parameters including central tendency (e.g. means) or other basic estimates (e.g. regression coefficient) AND variation (e.g. standard deviation) or associated estimates of uncertainty (e.g. confidence intervals)
☐	☒	For null hypothesis testing, the test statistic (e.g. *F*, *t*, *r*) with confidence intervals, effect sizes, degrees of freedom and *P* value noted<br>*Give P values as exact values whenever suitable.*
☒	☐	For Bayesian analysis, information on the choice of priors and Markov chain Monte Carlo settings
☒	☐	For hierarchical and complex designs, identification of the appropriate level for tests and full reporting of outcomes
☒	☐	Estimates of effect sizes (e.g. Cohen's *d*, Pearson's *r*), indicating how they were calculated

Our web collection on statistics for biologists contains articles on many of the points above.

Software and code

Policy information about availability of computer code

Data collection	Electrophysiological data was acquired with Patchmaster (HEKA Elektronik, Lambrecht, Germany) at 5 kHz and filtered with Frequency Devices 8-pole Bessel filter at a corner frequency of 2 kHz. Publicly available software packages SHAKE algorithm and NAMD were used for the simulations (https://www.ks.uiuc.edu/Research/NAMD).
Data analysis	Analysis of electrophysiological data was performed using Ana (M. Pusch, Istituto di Biofisica, Genova), Sigmaplot 10.0 (SPSS Inc.) and Prism 8.0 (GraphPad, San Diego, CA, USA). A one sided Student's t-test with a Bonferroni correction was used to evaluate statistical significance. Publicly available software package VMD was used for the analysis and visualization of the molecular system (https://www.ks.uiuc.edu/Research/vmd/).

For manuscripts utilizing custom algorithms or software that are central to the research but not yet described in published literature, software must be made available to editors and reviewers. We strongly encourage code deposition in a community repository (e.g. GitHub). See the Nature Portfolio guidelines for submitting code & software for further information.

Data

Policy information about availability of data

All manuscripts must include a data availability statement. This statement should provide the following information, where applicable:

- Accession codes, unique identifiers, or web links for publicly available datasets
- A description of any restrictions on data availability
- For clinical datasets or third party data, please ensure that the statement adheres to our policy

All constructs and electrophysiological traces are available on request.

Human research participants

Policy information about studies involving human research participants and Sex and Gender in Research.

Reporting on sex and gender	N/A
Population characteristics	N/A
Recruitment	N/A
Ethics oversight	N/A

Note that full information on the approval of the study protocol must also be provided in the manuscript.

Field-specific reporting

Please select the one below that is the best fit for your research. If you are not sure, read the appropriate sections before making your selection.

☒ Life sciences ☐ Behavioural & social sciences ☐ Ecological, evolutionary & environmental sciences

For a reference copy of the document with all sections, see nature.com/documents/nr-reporting-summary-flat.pdf

Life sciences study design

All studies must disclose on these points even when the disclosure is negative.

Sample size	Electrophysiological measurements were repeated 4+ times from 3+ independent oocyte batches to ensure that multiple independent biological samples were tested.
Data exclusions	All constructs were tested side by side with control preparations of the corresponding WT channel and of mock-injected oocytes. In some rare cases the control samples behaved anomalously, judged by parameters (current magnitude and/or reversal potential) outside 3 times the standard deviation of the mean for the wildtype. In these cases, the whole batch of oocytes was disregarded.
Replication	Electrophysiological measurements were repeated 4+ times from 3+ independent oocyte batches. All attempts to replicate/reproduce experiments were successful.
Randomization	Randomization was not relevant to the design of the functional electrophysiological experiments as no grouping was involved in the experiments.
Blinding	The experimental design did not require blinding for electrophysiological experiments as a defined system was used in all cases.

Reporting for specific materials, systems and methods

We require information from authors about some types of materials, experimental systems and methods used in many studies. Here, indicate whether each material, system or method listed is relevant to your study. If you are not sure if a list item applies to your research, read the appropriate section before selecting a response.

Materials & experimental systems

n/a	Involved in the study
☒	☐ Antibodies
☒	☐ Eukaryotic cell lines
☒	☐ Palaeontology and archaeology
☒	☐ Animals and other organisms
☒	☐ Clinical data
☒	☐ Dual use research of concern

Methods

n/a	Involved in the study
☒	☐ ChIP-seq
☒	☐ Flow cytometry
☒	☐ MRI-based neuroimaging
